# Can ploidy levels explain the variation of *Herbertia lahue* (Iridaceae)?

**DOI:** 10.1590/1678-4685-GMB-2023-0137

**Published:** 2024-08-23

**Authors:** Eudes Maria Stiehl-Alves, Ariane Tonetto Vieira, Caroline Trevelin, Alexandre Cristante Martins, Tatiana Teixeira de Souza-Chies, Eliane Kaltchuk-Santos

**Affiliations:** 1 Universidade Federal do Rio Grande do Sul, Instituto de Biociências, Programa de Pós-Graduação em Botânica, Porto Alegre, RS, Brazil.; 2 Universidade Federal do Rio Grande do Sul, Instituto de Biociências, Programa de Pós-Graduação em Genética e Biologia Molecular, Porto Alegre, RS, Brazil.; 3 Universidade Federal do Rio Grande do Sul, Instituto de Biociências, Departamento de Genética, Porto Alegre, RS, Brazil.

**Keywords:** CMA/DAPI, FISH, genome size, morphometry, pollen analysis, polyploidy

## Abstract

Polyploidy is often related with phenotypic variation, as observed in *Herbertia lahue*, a geophyte species. This study examined the *H. lahue* polyploid series and departure in cytogenetic, morphometric, and pollen data. Diploids (2*n*=2*x*=14) present bimodal karyotype with two long and five short chromosome pairs, while hexaploids (2*n*=6*x*=42) and octoploids (2*n*=8*x*=56) present a gradual decrease in chromosome size. All cytotypes have CMA^+^/DAPI^-^ bands co-localized with 18S rDNA sites in the satellite region (no DAPI^+^ bands in any cytotype). Unlike diploids and octoploids, 5S rDNA interstitial sites in hexaploids are not in a syntenic position with 18S rDNA sites. Genome size is effective as an indirect predictor of the cytotypes since 2C-values increased according to ploidy level. The reduction in the number of the rDNA sites in polyploids associated with their lower 1C*x*-values compared to diploids may suggest a genome downsizing process. Morphometric analysis revealed significant differences among cytotypes, and discriminant analysis identified three morphometric groupings corresponding to the cytotypes. The phenotypic variation observed in pollen grains, bulbs, and ovary characters suggested the gigas effect. Concluding, remarkable differentiation was observed at both genomic and phenotypic characters in all the cytotypes analyzed, suggesting a possible ongoing speciation process in *H. lahue*.

## Introduction

Polyploidy (or whole genome duplication) has been considered an important mechanism involved in plant adaptation and speciation, being a key event in the evolution of Angiosperms (Fox et al., 2020; Van de Peer et al., 2021). Polyploidy can drastically change phenotypic attributes or ecological preferences in a few generations (Rice et al., 2019; Van Drunen and Husband, 2019; Fox *et al*., 2020; Rezende et al., 2020; Van de Peer *et al*., 2021). Understanding the mechanisms behind phenotypic plasticity along with possible adaptive and ecological changes can bring some light about the diversification and evolution of a taxa (Zenil-Ferguson et al., 2017; Fox *et al*., 2020; Van de Peer *et al*., 2021).

Polyploidy plays a remarkable role in the evolution of the family Iridaceae resulting in chromosome numbers ranging from *2n* = 6 to 230 (Goldblatt and Takei, 1997; Goldblatt and Manning, 2008; Souza-Chies et al., 2012; Moraes et al., 2015). Neopolyploidy (relating to infrageneric polyploidy) is common in members of the family from the Northern Hemisphere (Goldblatt and Takei, 1997). More recent studies showed that several genera of Iridoideae from South and Central America present intrageneric and intraspecific polyploid series (Moreno et al., 2009; Alves et al., 2011; Tacuatiá et al., 2012; Moraes *et al.,* 2015; Tacuatiá *et al*., 2016; Burchardt et al., 2018). Although chromosome number and genome size are valuable characters in the circumscription of several genera of Iridaceae, cytogenetic information for South American species are still limited (Souza-Chies *et al*., 2012; Moraes *et al*. 2015). 

The herbaceous *Herbertia* Sweet comprises eight species of geophytic, insect-pollinated plants with few leaves, usually with violet flowers presenting free and unequal tepals (Goldblatt and Manning, 2008). *Herbertia lahue* (Molina) Goldblatt is a species that has a wide geographic distribution and can be found in grasslands of the south of the Neotropical region, including Brazil, Argentina, Uruguay, Paraguay, and Chile. As in other species of *Herbertia*, flowers are the foremost source of characters for recognition of *H. lahue*, but the morphometric variation in floral characters is significant and extensive (Stiehl-Alves et al., 2016), making species boundaries questionable. The taxonomic history of *H. lahue* is complex and subject to debate, a result of uninformative descriptions based on herborized material, an inefficient method of preserving the floral characters of Iridaceae. Historically, three subspecies were accepted (Goldblatt, 1977), and a recent taxonomic study suggested the division of *H. lahue* into three species (Deble, 2021). However, this latter used few morphological traits to recognize the species besides some overlapping characters. Given the evolutionary complexity and issues highlighted by a previous study (Stiehl-Alves *et al*., 2016), we accept the understanding of Plants of the World Online (2023), which considers that the three species are synonymous with *H. lahue*, at least until the species boundary is checked considering different species concepts. 

Like other genera of clade A of Tigridieae, *H. lahue* has the basic number *x* = 7 (Moraes et al., 2015) with four ploidy levels (2*x,* 4*x,* 6*x* and 8*x*) reported (Winge, 1959; Baeza et al., 2001; Moreno et al., 2009; Moraes *et al*., 2015; Stiehl-Alves et al., 2016; Martins et al., 2021). However, recent investigations were unable to find tetraploid plants (Moreno *et al*., 2009; Moraes *et al*., 2015; Stiehl-Alves *et al*., 2016; Martins *et al*., 2021; Vieira et al., 2023). Over the years working with this species, we have verified a set of floral characters that allow us to recognize each cytotype in the field. A previous study investigated the morphometric variation in floral characters of *Herbertia lahue* polyploids and observed significant differences in the measurements of the outer and inner tepals, length of the staminal column, anthers, and ovaries (Stiehl-Alves *et al*., 2016). Despite the statistical differences observed in that study, hexaploids and octoploids formed two partially overlapping phenetic groups and lacked comparisons with diploid *H. lahue*. Thus, a more detailed morphometric analysis including other floral and bulb features, as well as diploid plants, is important to clarify the possible effects of polyploidy on the phenotypic diversity of *H. lahue*.

This study employed cytogenetic and morphometric approaches to investigate the existence of distinct morphological groups in *H. lahue* and their possible relationship with ploidy level aiming to contemplate the effects of polyploidy on the polymorphism of this species.

## Material and Methods

### Plant material

**Figure 1 -:**
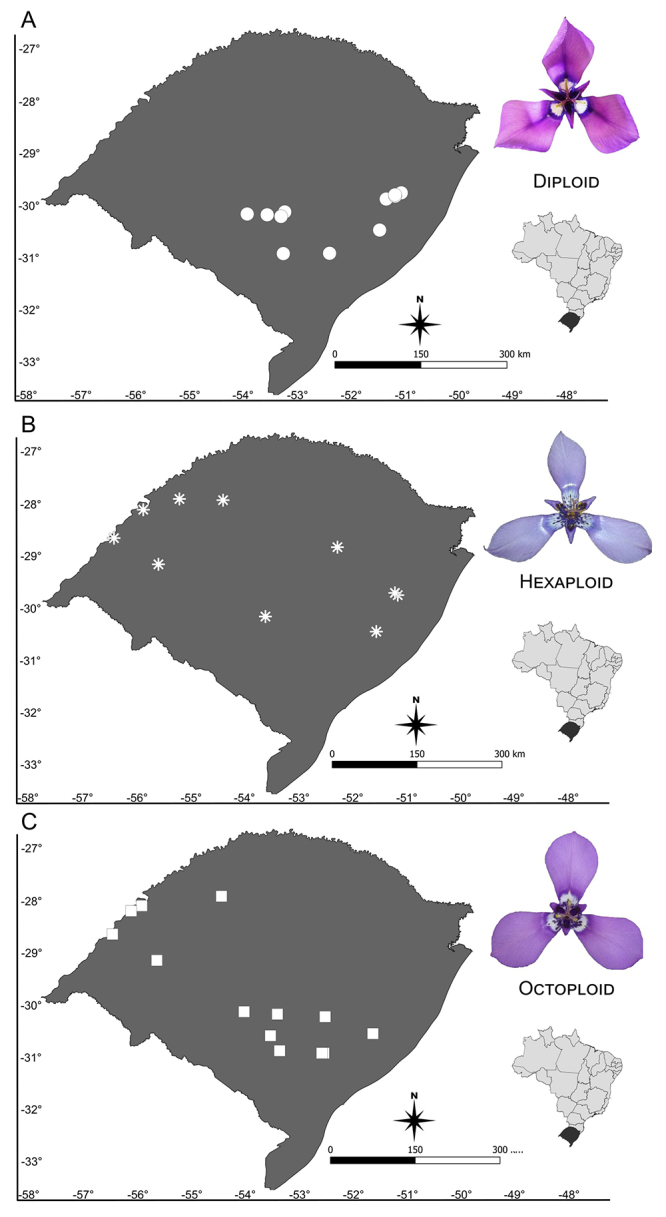
Map with the locations of *Herbertia lahue* populations sampled in this study. Circles indicate diploid populations, asterisks indicate hexaploid populations and squares indicate octoploid populations.

Twenty-eight populations of *Herbertia lahue* were sampled *in situ* during the flowering months (October and November) in 2018 and 2019 across southern Brazil (Figure 1; Table S1) aiming the cytogenetic and morphometric studies. The collection effort covered a representative part of the geographic distribution of *H. lahue*, to confirm the existence of all cytotypes mentioned in the literature. Samples were collected from living plants from various individuals spaced at least 5 m apart to minimize the possibility of resampling clonal individuals, as the species also propagates vegetatively by bulb fragmentation. Bulbs of five to 10 individuals per population were planted and cultivated in the experimental garden of the Instituto de Biociências of Universidade Federal do Rio Grande do Sul (UFRGS) for cytogenetic analyses. Collected flowers were preserved in glycerol 3:7 ethanol (at least 10 samples per population) for morphological analyses. Furthermore, one specimen from each population was incorporated into the UFRGS herbarium (ICN).

### Cytogenetic analyses and flow cytometry

Chromosome counts were made using at least five individuals from each population (Table S1). Root tips were pretreated with 2 mM solution of 8-hydroxyquinoline for 4 h at 25 ºC and fixed in ethanol: acetic acid solution (3:1, v/v). Samples were digested in an enzymatic pool (2% cellulase - C1184 Sigma^®^ and 1% macerozyme - R10 Kinki Yakult MFG diluted in 20% pectinase E6287 - Sigma^®^) and slides were prepared by squashing the digested root tip in a drop of 45% acetic acid under a coverslip. After staining with 2% Giemsa, images of prometaphases and metaphase images were captured using a digital video camera coupled to a Zeiss Axioplan Universal microscope. For chromosome measurements, the software KaryoMeasure (Mahmoudi and Mirzaghaderi, 2023) was used, calibrating with the original scales of the selected images.

Chromosomes banding followed the protocol of Schweizer (1980) using chromomycin A_3_ (CMA_3_) and 4’,6-diamidino-2-phenylindole (DAPI) with modifications in staining time: chromomycin for 1.5 h, followed by DAPI for 45 min. Metaphases were analyzed in the fluorescence microscope Olympus BX51 (Olympus Co., Tokyo, JP) coupled with a DP72 digital camera and imaging software DP2-BSW (Olympus Co.). For FISH experiments, the slides stained with CMA/DAPI were discolored in ethanol:glacial acetic acid (3:1; v:v) for 30 minutes at room temperature under agitation and then dehydrated in an alcoholic series: 70 °C and 100 °C for 5 minutes each. Probes for the 18S and 5S ribosomal genes were used. For the rDNA probes, the D2 probes, a 500 bp fragment containing the *Lotus japonicus* ribosomal DNA gene (Pedrosa et al., 2002), and the R2 probe, a 6.5-kb fragment containing the rDNA region 18S-5.8S-25S from *Arabidopsis thaliana* (L.) Heynh. (Wanzenböck et al., 1997), were used to localize the 5S and 18S ribosomal DNA genes, respectively. Probes were labeled by nick translation using digoxigenin-11-dUTP (Life Technologies) in D2 labeling, and biotin-14-dATP (Roche) in R2. The digoxigenin-labeled probe was detected with anti-digoxigenin linked to Rhodamine (Roche), while the biotin-labeled probe was detected with avidin-FITC (Sigma). FISH experiments were conducted according to Schwarzacher and Heslop-Harrison (2000), with some modifications. Slides were counterstained and mounted in Vectashield medium containing DAPI and observed using an epifluorescence microscope Olympus BX51 as previously described.

Flow cytometry was used to estimate genome size and infer ploidy level in ten populations of which at least five individuals per population were analyzed (Table S1). *Solanum lycopersicum* L. (2C = 1.96 pg) and *Pisum sativum* L. (2C = 9.09 pg) were used as internal standards. Young leaf fragments of 2 cm² were cut into Petri dishes containing 0.5 mL of LB01 nuclear extraction buffer. The suspension was adjusted to 1 mL using the same buffer, filtered through a 50 μm nylon mesh in the microtube. Subsequently, suspensions were stained in the dark with propidium iodide (Sigma^®^) simultaneously with RNase, both at 50 µg mL ^1^ (Doležel et al., 2007). Samples were analyzed on a BD FACSAria™ III flow cytometer equipped with two excitation lasers 488 nm 20 mW and 640 nm 17mW of power. Flow cytometry statistics and histograms were generated in BD FACSDiva version 6.1.3 software. Ploidy screening and total nuclear DNA content (2C) were assessed by relative computation assuming a linear relationship between fluorescent signals from target-stained nuclei and its internal standard using the formula proposed by Galbraith et al. (1998). Monoploid genome size (1C*x*) was also estimated to represent the DNA content in a basic chromosome set (*x*) of a somatic cell (Greilhuber et al., 2005). Only measurements with coefficients of variation (CV) less than 5% were considered. Statistical analysis to verify differences in DNA content ploidy levels of *H. lahue* was performed in R version 4.0.5 (R Core Team, 2021).

### Pollen grain analyses

The analyses of pollen grains were carried out in at least three individuals for each nine populations (Table S1). Samples were collected before anthesis and fixed in the Carnoy solution. Anthers with approximately 5-8.5 mm in size were macerated in 1% Alexander solution (Alexander, 1980). Given the apparent difference in pollen production between the different cytotypes, the anthers from hexaploids and octoploids were macerated in 200μL of 1% Alexander solution, while those from diploids were macerated in 1mL. Samples of 20 μL were transferred to the Neubauer Chamber for pollen grains counting under Zeiss Axioplan optical microscope. A total of four repetitions per individual were performed.

To estimate the viability of pollen grains, they were stained in 1% Alexander and evaluated immediately after staining. A total of 9,000 grains for the diploid cytotype (18 individuals), 8,000 grains for the hexaploid (16 individuals) and 4,500 grains for the octoploid cytotype (18 individuals) were analyzed. The grains were classified into viable and non-viable (Alexander, 1980), where the former are filled purple color, while the latter remain empty and colored green. The pollen size was also investigated and polar (P) and equatorial (E) axes of 20 viable pollen grains were measured using the AxioVision Zeiss software for each sampled individual, with a repetition of three individuals for each population. 

Using the R version 4.0.5 (R Core Team, 2021), analyzes of the statistical significance of the variation in size, amount and pollen viability of pollen grains for the three cytotypes of *H. lahue* were carried out. The variation of grain measurements was also tested among populations of the same cytotype. The Shapiro-Wilk test and the Levene test were used to verify the normality and homogeneity of variances. For parametric data, One-Way ANOVA with post-hoc Tukey’s HSD test was used to compare the means of the groups. Non-parametric data were analyzed using the Kruskal-Wallis test, followed by the Dunn test with Bonferroni correction.

### Morphological analysis

For the morphometric analysis, 17 floral and three vegetative characters were measured with digital calipers (Table S2 and Figure S1). The morphological terminology is in accordance with Goldblatt and Manning (2008) and Beentje (2010). Univariate statistics and box plots were used to examine variation of characters among cytotypes and Shapiro’s test was applied to check for normal distribution. One-way ANOVA (for normally distributed data) or Kruskal-Wallis test (non-normally distributed data) were used to examine which morphological traits vary among diploids, hexaploids and octoploids of *H. lahue*, and post-hoc Tukey test or Wilcoxon rank sum test were used to check for differences. Levels of significance were *P* > 0.05: not significant (n.s.); *P* ≤ 0.05: significant (*); *P* ≤ 0.01: very significant (**); *P* ≤ 0.001: highly significant (***). These statistics were computed in R version 4.0.5 (R Core Team, 2021). Discriminant analysis was estimated in PAST 4.06 (Hammer et al., 2001) using a dataset with 18 morphological characters with significant differences (Table S2) to examine the overall pattern and morphological differentiation in *H. lahue*. The Mahalanobis distance was calculated from the pooled within-groups (groups = cytotypes) covariance matrix, giving a linear discriminant classifier. Group assignment was cross-validated by Jackknifing procedure and missing data were supported by column average substitution procedure. The biplot option was selected to display variables on the scattergraph.

## Results

### Chromosome number, karyotype and genome size

**Figure 2 -:**
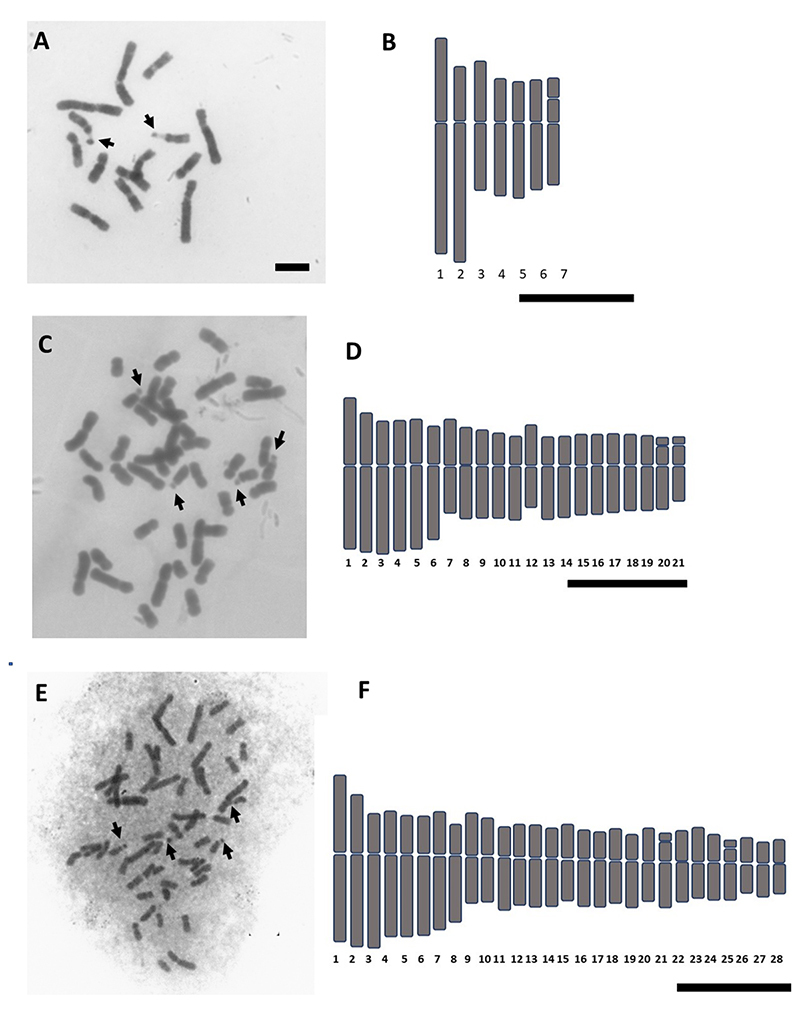
Mitotic metaphases of each cytotype of *Herbertia lahue* and its respective ideogram. (A) and (B) Diploid *H. lahue;* (C) and (D) Hexaploid *H. lahue;* (E) and (F) Octoploid *H. lahue.* Bars = 5 μm.

Chromosome counts confirmed the occurrence of three cytotypes in *H. lahue,* each of which corresponds to a specific morphotype. Seven populations are diploids (2*n* = 2*x* = 14); five are hexaploids (2*n* = 6*x* = 42); and nine populations are octoploid (2*n* = 8*x* = 56). None of the investigated populations had tetraploid plants (Table 1, Figure 2). Diploid cytotypes exhibited bigger chromosomes (~6.22 µm) than polyploids, around 4 µm (Table 1 and Figure 2). All cytotypes presented asymmetric karyotypes with only metacentric and submetacentric chromosomes (Table 1; Figure 2). Additionally, the octoploid cytotype had a difference of almost six times between the largest and the smallest chromosome pairs. As expected, haploid chromosome length (HCL) increases with ploidy level (Table 1). The diploid is clearly bimodal with two long and five short chromosomes, while polyploids have their chromosome length decreasing gradually (Figure 2). The karyotypic formula of diploids is 2*n* = 4M + 10SM, with a satellite located on the short arm of chromosome pair 7 (Table 1 and Figure 2). The hexaploids have 2*n*= 26M + 16SM with two pairs of satellites while octoploids have 2*n*= 34M + 22SM and two pairs of satellites, also (Table 1 and Figure 2). (Figure 2).

**Table 1 -:** Summary of cytogenetic data of *Herbertia lahue* cytotypes.

Parameters	Ploidy levels		
	**2*n* = 2*x* = 14**	**2*n* = 6*x* =42**	**2*n* = 8*x* = 56**
Karyotypic formula (2*n*)	4M + 10SM	26M + 16SM	34M + 22SM
HCL (µm)	87.16	168.06	203.91
C (µm)	6.2	4.0	3.7
Size of the largest pair (µm)*	9.55	6.27	6.90
Size of the smallest pair (µm)**	4.49	2.59	1.16
Stebbins asymmetry category	1B	1B	2C
A2	0.31	0.28	0.34
Number of chromosomes with CMA^+^ bands	1 pair	2 pairs	3 pairs
Number of CMA bands	2 (ter)	4 (ter)	6 (ter)
Number of 35S sites	2 (ter)	4 (ter)	6 (ter)
Number of 5S sites	6 (int)	10 (int)	20 (int)
2C ± SD (pg)	4.33 ± 0.30 ^c^	12.28 ± 0.30 ^b^	16.3 ± 0.20 ^a^
1C*x* ± SD (pg)	2.17 ± 0.15 ^a^	2.05 ± 0.05 ^a^	2.04 ± 0.02 ^a^

^1^ M = metacentric chromosomes - SM = submetacentric chromosomes.

^2^ HCL = haploid chromosome length - C = mean chromosome length - A2 = interchromosomal asymmetry index.

^3^ * = average size of the largest chromosome pair - ** = average size of the smallest chromosome pair.

^4^ ter = terminal position - int = interstitial position.

^5^ 2C = Genome size expressed in mean - 1C*x* = monoploid genome size - SD = standard deviation.

6Data from 2C and 1Cx values were analysed by one-way ANOVA (p ≤ 0.001) and different letters represented significant differences between means by Tukey’s test.

Flow cytometry procedure resulted in histograms with coefficients of variation below 5%. Statistical analysis of genome size was performed for each cytotype of *H. lahue* and 2C-values and 1C*x*-values followed a normal distribution tested by Shapiro-Wilk test. Differences were observed in 2C-values between cytotypes (Table 1), but no differences within cytotypes (*F* = 2223, df = 2, *p*-value < 0.000). Considering the DNA content represented by the monoploid 1C*x*-values, polyploid samples have lower values than diploids (Table 1), although these differences are not statistically significant (*F*= 2.357, df = 2, *p*-value = 0.145). 

**Figure 3 -:**
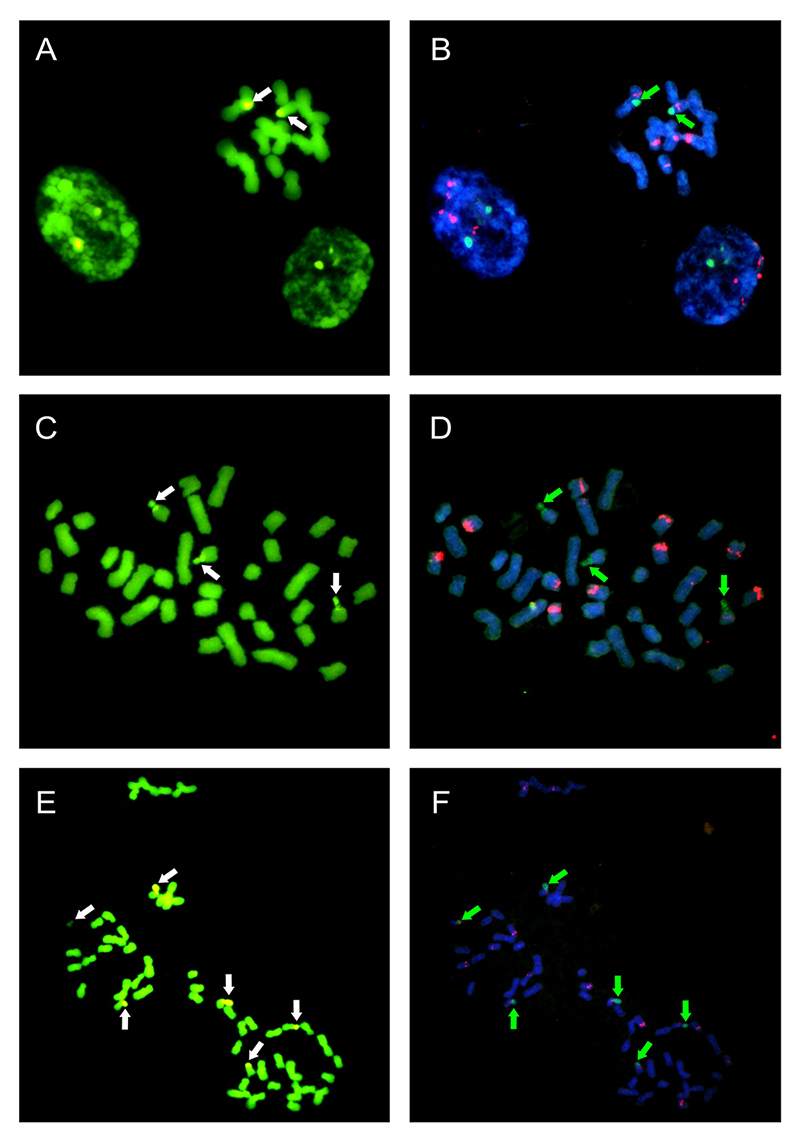
Mitotic metaphases of *Herbertia lahue* with CMA/DAPI chromosome banding (CMA^+^ bands yellow) and hybridized with 18S (green) and 5S (red) rDNA probes. (A) and (B) Diploid *H. lahue*; (C) and (D) Hexaploid *H. lahue;* (E) and (F) Octoploid *H. lahue*. Arrows indicate 18S rDNA sites co-located with CMA bands. Bar = 5 μm.

Data of chromosome banding revealed terminal CMA^+^/DAPI^-^ bands located only in the secondary constriction and satellites of pair 7 (Figure 3 A ). No DAPI^+^ band was observed in any chromosome. The fluorescent *in situ* hybridization data indicate that 18S rDNA sites are always co-localized with CMA^+^ bands (Figure 3 B ). Four chromosome pairs, including the pair 7, have interstitial sites of 5S rDNA revealed as a pair of dot-like sites. CMA banding and FISH techniques applied in hexaploids showed once again co-localization of GC rich bands (CMA^+^) and 18S rDNA sites in the two chromosome pairs with satellites (Figures 3 C  and 3D). Unlike diploids and octoploids, these chromosomes in hexaploids do not exhibit 5S rDNA sites in a syntenic position with 18S rDNA sites. Nevertheless, the dot-like sites are found in five other chromosome pairs, always in the interstitial region. The differential staining of octoploid cytotype showed three pairs of chromosomes presenting terminal CMA^+^ associated with 18S rDNA (Figures 3 E  and 3F), although only two pairs of satellite chromosomes were visualized by conventional staining. These three chromosomes also carry the dot-like sites of 5S rDNA interstitial, as do seven other pairs of chromosomes.

### Pollen analysis

**Figure 4 -:**
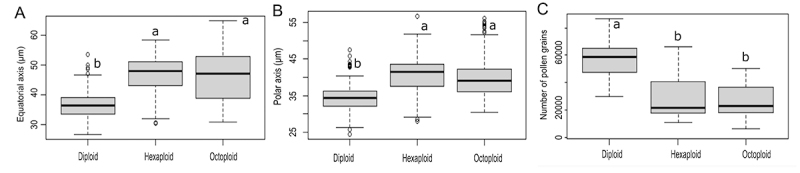
Effect of ploidy level on equatorial axis and polar axis measurements, and on the quantity of pollen grains per anther in *Herbertia lahue* cytotypes. ^a, b^ Letters indicate differences and mean values marked with the same letter are not significantly different at *P* < 0.05, by Tukey’s test.

Pollen analyses highlighted that there are significant differences for the size (equatorial axis: *H* = 344.56, *P* < 0.001; polar axis: *H* = 284.9, *P* < 0.001) and amount of pollen grains (*F* = 24.27, *P* < 0.001), but differences are non-significant for pollen viability (*H* = 1.3073, *P* = 0.5201) of cytotypes. Tukey’s test revealed significant differences between diploids and polyploids in equatorial and polar axis measurements, as well as in the number of pollen grains (*P* < 0.05). However, there were no significant differences between hexaploids and octoploids for these three parameters (see Figure 4 and Table S3). The highest means for equatorial and polar axis measurements were observed in polyploid samples (Figure 4A and 4B), while diploids have both shortest axes, but with a significantly greater number of pollen grains. (Figure 4 C ). Pollen grain viability was substantial (above 90%) for the three cytotypes (Table S3). 

### Morphometry and multivariate analysis

**Figure 5 -:**
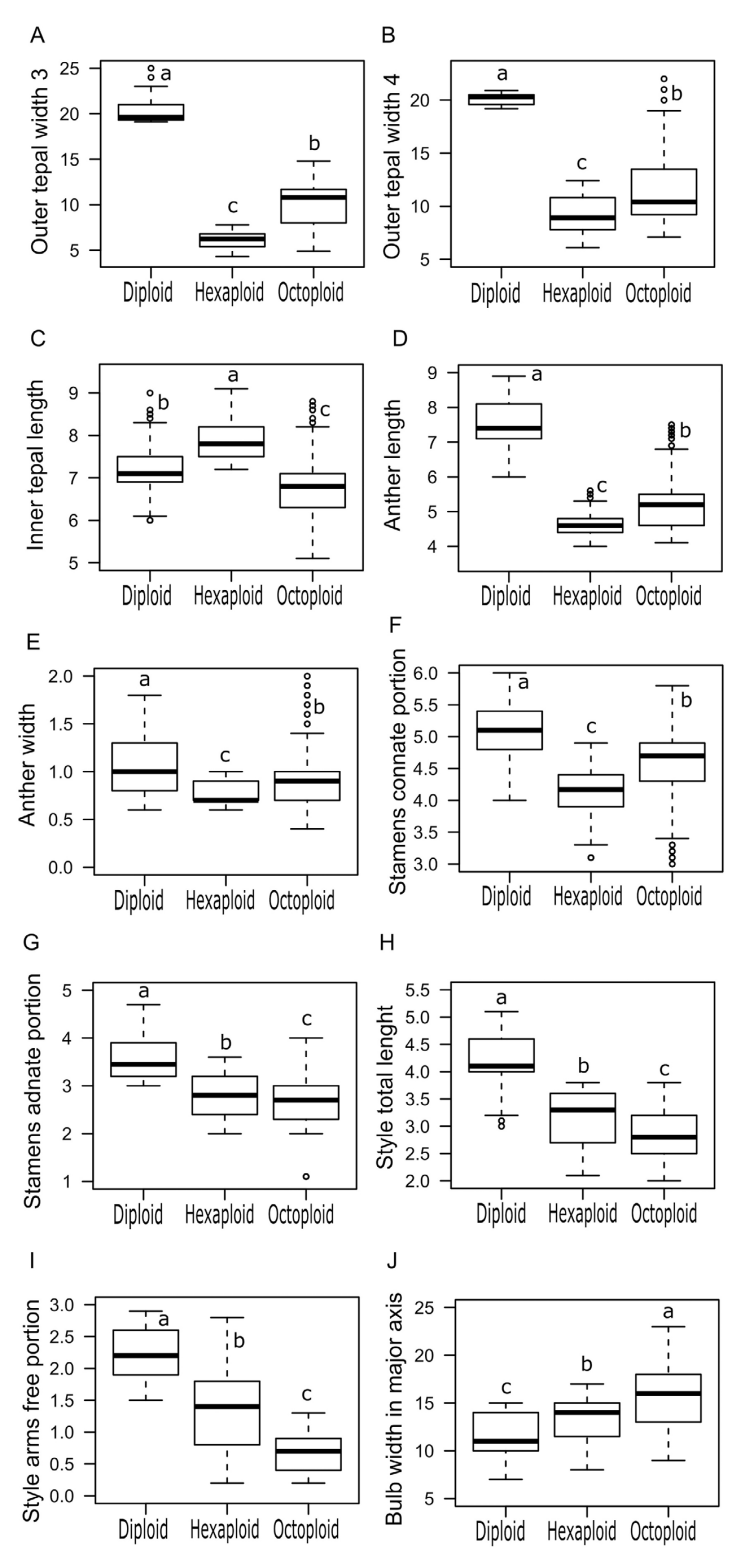
Effect of ploidy level on the morphometry of characters examined in *Herbertia lahue* cytotypes. ^a, b, c^ Letters indicate differences and mean values marked with the same letter are not significantly different at *P* < 0.05, by Tukey’s test.

The morphometric analysis identified significant differences in 18 of 20 characters studied in samples representing the three cytotypes (Table S2), showing that *H. lahue* is a species with noticeable morphological variation. The post-hoc Wilcoxon rank sum test identified significant differences that distinguish each cytotype from the others in ten characters (Figure 5), of which nine are floral traits and one is an underground bulb measurement (bulb width in major axis). Among the nine floral traits that distinguish each of the *H. lahue* cytotypes, four correspond to androecium traits (anther length, anther width, stamens connate portion and stamens adnate portion) and two gynoecium characters (style total length and style arms free portion).

**Figure 6-:**
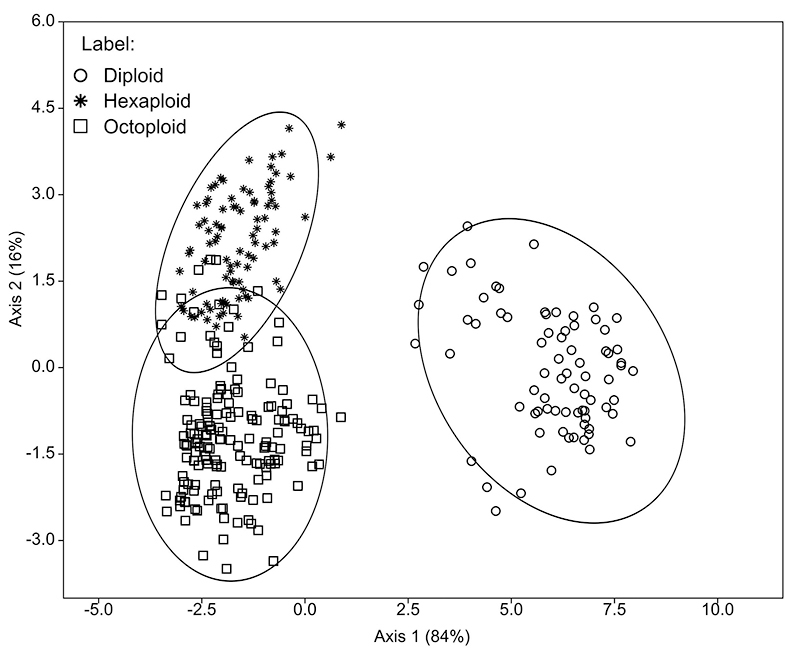
Discriminant analysis performed with 18 morphological characters examined in *Herbertia lahue* cytotypes.

Discriminant analysis using 18 morphological characters with significant differences identified three clusters with 94.85% cross-validated by Jackknifing group assignment. In the discriminant analysis, eigenvalues of the first and second canonical variables were found to be 10.597 and 1.9797, explaining 84% and 16% variation respectively, among the samples from the three cytotypes of *H. lahue* analyzed (Figure 6). The first axis of the scatterplot discriminated a morphometric cluster with positive eigenvalues containing exclusively diploid samples (100% correctly assigned). The second axis of the scatterplot discriminated two partially overlapping morphometric groups containing, respectively, hexaploid samples (85% correctly set) with positive eigenvalues, and octoploid samples (99% correctly assigned) with positive and negative eigenvalues (Figure 6). In the discriminant analysis, variables with higher positive and negative scores on axis 1 and axis 2 are related to measures of outer and inner tepals, as well as androecium and gynoecium traits (Table 2). 

**Table 2 -:** Variable loadings in the discriminant analysis of 18 morphometric characters analyzed in *Hebertia lahue* cytotypes.

Variables	Axis 1 eigenvalues	Axis 2 eigenvalues
Outer tepal width 3	1.2344	-1.2252
Outer tepal width 4	1.0042	-0.7006
Outer tepal length	0.7040	0.0911
Anther length	0.3221	-0.2109
Syle arms free portion	0.1707	0.1862
Style total length	0.1691	0.1007
Style arms total length	0.1404	0.0001
Stamens adnate portion	0.0975	0.0429
Stamens connate portion	0.0748	-0.1275
Outer tepal width 2	0.0240	-0.1488
Anther width	0.0235	-0.0458
Inner tepal length	0.0056	0.3290
Inner tepal width	-0.0112	0.1686
Ovary width	-0.0228	-0.0108
Ovary length	-0.1413	-0.1048
Bulb length	-0.2030	-0.4169
Bulb width in minor axis	-0.2185	-0.3440
Bulb width in major axis	-0.2382	-0.3027

## Discussion

### Polyploidy in *Herbertia lahue*: Cytogenetic aspects

Species comprising polyploid series constitute a valuable material for studying the effects of genome duplication on phenotypic diversity and its implications for the establishment and maintenance of polyploid cytotypes (Van Drunen and Husband, 2019; Fox et al., 2020; Van de Peer et al., 2021). Intraspecific polyploidy has been reported in *H. lahue* including four ploidies: 2*x,* 4*x,* 6*x* and 8*x* (Winge, 1959; Kenton and Heywood, 1984; Goldblatt and Takei, 1997; Moreno et al., 2009; Moraes et al., 2015; Martins et al., 2021). Furthermore, a great morphological variation has been observed in the field, which was partially documented by Stiehl-Alves et al. (2016). Although *H. lahue* is an interesting model for polyploidy studies, there are several aspects that need to be investigated within an integrative approach.

In the present study we partially corroborate the previous data, since among the populations of *H. lahue* investigated we found diploid, hexaploid and octoploid cytotypes, but no tetraploids despite the large number of populations analyzed. The tetraploid chromosome number 2*n* = 28 was reported by Goldblatt (1982) and Goldblatt and Takei (1997) based on the work of Winge (1959). In fact, Winge (1959) reports the species as *Alophia amoena* (Griseb.) Kuntze, *Alophia* sp. and *A. pulchella* (Sweet) Kuntze, whose numbers would be 2*n* = 14; *n* = 15 + 5 and *n* = 15, respectively. Based on the images presented in Winge’s article, it appears to have been a mistake in identifying the species. The flower that is depicted as *A. amoena* seems to be *Herbertia pulchella* Sweet flower instead, as its outer tepals have a characteristic longitudinal white stripe, which is a diagnostic trait of *H. pulchella.* The apparent lack of tetraploids in the polyploid series of *H. lahue* is quite curious and raises several questions that will not be discussed at this time, as they are beyond the objectives of this study.

Moreno et al. (2009) cytogenetically analyzed three *Herbertia* species: *H. darwinii* Roitman and J.A. Castillo (diploid)*, H. quareimana* Ravenna (tetraploid) and *H. lahue* (hexaploid and octoploid). Taking into account the data reported in this study and our results, we can observe that *Herbertia* species share karyotypic features, with bimodality in the diploid species and a gradual reduction in the chromosome size of polyploids. In addition, all species have only metacentric and submetacentric chromosomes, where diploids have one pair of satellite chromosomes and polyploids have two pairs. The same karyotypic formula was found for the hexaploid cytotypes of *H. lahue* in our study and by Moreno *et al*. (2009): 26 M + 16 SM. However, for octoploids they reported 36M + 20 SM, while we found 34M + 22 SM, however, this discrepancy is possibly just a technical artifact. 

In fact, these karyotypic characteristics of *Herbertia* species are quite conserved within the Tigridieae tribe and have been widely described in other genera, especially those of clade A (Goldblatt, 1982; Kenton and Heywood, 1984; Alves et al., 2011; Moraes et al., 2015). Bimodal karyotypes have already been reported by our team and other studies as an important evolutionary trait, and having been described for the diploid *Herbertia furcata* (Klatt) Ravenna (Moraes *et al.*, 2015), as for other genera like *Alophia, Calydorea, Cipura* and *Eleutherine* (Goldblatt and Takei, 1997; De Tullio et al. 2008; Alves et al., 2011; Moraes et al., 2015; Martínez et al., 2017; Baéz et al. 2019). The conserved base number *x* = 7 and the bimodal karyotype with two large and five small chromosome pairs are characteristics that support the monophyly of the tribe (Goldblatt, 1982; Goldblatt and Takei, 1997; Moraes *et al*., 2015).

Cytogenetic analyses including fluorescent chromosome banding and *in situ* hybridization of rDNA sites have been widely used and provide important information regarding karyotypic evolution and differentiation of evolutionary lineages (Guerra, 2012; Moraes et al., 2015). Nevertheless, as far as we know, there are few cytogenetic studies using these approaches for Tigridieae species (Feitoza and Guerra, 2011; Alencar et al., 2018; Arroyo-Martínez et al., 2018; Felix et al., 2019; Baéz et al., 2019; Arroyo-Martínez *et al*., 2020) and only one for *Herbertia* (Moreno et al., 2009). Here we bring some preliminary results of CMA/DAPI differential staining and FISH with 18S and 5S rDNA probes. All cytotypes have CMA^+^/DAPI^-^ bands occurring exclusively in those chromosomes that anchor the satellites associated with the NOR region. Although the three cytotypes differ in some aspects, they are similar in the absence of AT-rich regions, since DAPI+ bands were not observed in any chromosome of the complement. The fluorochromes CMA and DAPI bind preferentially to GC-rich and AT-rich regions, respectively (Schweizer, 1976; Guerra, 2000; Barros and Guerra, 2010). Thus, the non-observation of DAPI bands may be related to the absence of long AT-rich sequences forming blocks and making their detection difficult by fluorochrome banding. 

The satellite chromosomes present 18S rDNA sites colocalized with CMA bands in terminal position, while the sites of 5S rDNA are always interstitial looking a dot-pair. Our study revealed through CMA/DAPI banding that *H. lahue* is poor in heterochromatic regions which are restricted to GC rich sequences associated with the nucleolar organizer region. 

The same distribution pattern of CMA/DAPI bands and 18S and 5S rDNA sites was observed by Moreno et al. (2009) for the hexa and octoploid cytotypes of *H. lahue*, as well as for *H. darwinii* and *H. quareimana*. This pattern seems to be characteristic of *Herbertia* species, and is not shared by other genera of the tribe. For example, unlike *Herbertia*, *Calydorea crocoides* Ravenna, a species belonging to the same clade of *Herbertia* within Tigridieae, presents DAPI^+^ bands on pericentromeric position of all chromosome pairs (Alencar et al., 2018). Likewise, proximal DAPI^+^ bands are found in all chromosomes of *Eleutherine bulbosa* (Mill.) Urb. (Feitoza and Guerra, 2011) while *Alophia drumondii* (Graham) R.C.Foster presents punctate DAPI bands in the pericentromeric region of a unique small chromosome pair (Felix et al., 2019). Regarding rDNA sites, *E. bulbosa* and *E. latifolia* (Standl. and L.O. Williams) Ravenna have a single chromosome pair containing both the 35S and 5S sites (Baéz et al., 2019) while *Tigridia pavonia* (L.f.) Redouté has three pairs of chromosomes with both genes, in addition to individual sites in other chromosomes (Arroyo-Martínez et al., 2018, 2020). *Herbertia lahue* diploids and octoploids have also 5S and 18S rDNA sites in synteny position, in their satellite chromosome, but on the other hand, the hexaploids do not have any signal of 5S rDNA sites in these chromosomes. Intraspecific variation in the number and position of sites in polyploid cytotypes is not rare and suggests genomic reorganization, but can lead to erroneous taxonomic interpretations (Guerra, 2012; Chiavegatto et al., 2019). Perhaps the lack of sites in the two satellite pairs indicate the existence of karyologically distinct evolutionary lineages.

The karyotypic features of *H. lahue* cytotypes follow a trend described by  Roa and Guerra (2012) for Angiosperms, with predominance of one to two 45S rDNA sites per haploid complement, occurring preferentially in the short arm, in regions rich in GC. On the other hand, although most species and genera contain a single pair of 5S rDNA sites (Guerra, 2012), in *H. lahue*, the number of sites varies from 6 to 20, in diploids and octoploids, respectively. Moreover, considerations need to be made regarding the number of CMA+ bands and 18S and 5S rDNA sites presented by the different cytotypes of *H. lahue*, since they do not follow the expected increase according to the ploidy level. In fact, considering the monoploid complement, there is a pronounced reduction in these regions in both polyploid cytotypes compared to the diploid one (see Table 1). A similar pattern was reported in *Sisyrinchium micranthum* Cav., a species from the subfamily Iridoideae, where a substantial reduction in the number of 35S rDNA sites was observed in polyploids compared to diploids, as well as a reduction in genome size (Tacuatiá et al., 2016). In intraspecific cytotypes of recent divergence, there is usually maintenance of the number and pattern of bands and/or sites per monoploid complement (Rieseberg and Willis, 2007; Berjano et al., 2009; Roa and Guerra, 2012; Cordeiro et al., 2022). In the case of autopolyploids, there is a proportional increase in these regions according to the ploidy level. On the other hand, intrageneric polyploid series have a longer divergence time and usually have a reduction in the number of sites compared to their diploids. The loss of duplicated regions has been reported in several species (see the survey of Roa and Guerra, 2012) including other polyploids of Iridaceae (Tacuatiá *et al*., 2016). The whole genome duplication results in extensive changes, with chromosomal rearrangements, loss of regions and/or silencing of redundant genes (Leitch and Bennett, 2004; Soltis et al., 2004; Wendel et al., 2018).

Genome size estimates obtained here are in agreement with those of our previous studies (Moraes et al., 2015; Martins et al., 2021). These findings suggest that genome size can be used to indirectly infer ploidy level in *H. lahue* cytotypes, as in other angiosperms (Zozomová-Lihová et al., 2015; Visger et al., 2016). In the present study, the haploid chromosome length (HCL) and 2C-values increased according to ploidy level, as expected. Nevertheless, the diploid cytotype has larger chromosomes than the polyploid ones (greater average chromosome length) and higher 1C*x*-value which decreases as the ploidy level increases. Although the difference between the 1C*x-*values of diploids and polyploids is not statistically significant, together with the reduction of rDNA sites in polyploids, genome downsizing seems to be an ongoing evolutionary pathway that could allow polyploid plants to adapt to new ecological environments, since *H. lahue* diploid plants present a more restrict geographical distribution than the polyploid cytotypes (Leitch and Bennett, 2004; Soltis et al., 2004; Pellicer et al., 2018; Wang et al., 2021). Similar results were found by Tacuatiá et al. (2016), studying the polyploid series of *S. micranthum*. The authors found a significant difference in the 1C*x*-values of diploids in relation to polyploids, but there was no difference between tetraploids and hexaploids.

Genome downsizing has been reported in other Iridoideae genera (Tacuatiá et al., 2012; Moraes et al., 2015; Tacuatiá *et al*., 2016; Burchardt et al., 2018). Such genome structural reorganization by reduction of monoploid genome size is reported as a tendency toward genome downsizing for many plants, and is common in Iridaceae species presenting different cytotypes (Leitch and Bennett, 2004; Doležel et al., 2007; Pellicer et al., 2018). Reduction in genome size may occur in different proportions via auto- or allopolyploidization (Soltis et al., 2004). This is a widespread phenomenon of biological significance. The polyploidization is considered an evolutionary dead end (Mayrose et al., 2011). This is because the newly formed polyploid species in nature may be extinct in a few generations taking into account their fitness disadvantages. The neopolyploids face the minority cytotype exclusion (Levin, 1975), beside the low fertility in consequence of meiotic instability. The diploidization is a pathway to avoid the new polyploid extinction, in which genomic redundancy is removed, duplicated genes are lost resulting in a genome shrinkage. Such chromosome rearrangement is followed by bivalent pairing and disomic inheritance (Leitch and Bennett, 2004; Porturas and Segraves, 2020). From this scenario polyploidy arises as an important mechanism for plants diversification, adaptation and speciation (Wendel et al., 2018).

### The effect of polyploidy on pollen traits of *Herbertia lahue*

Frequently, polyploid organisms differ from diploids by remarkable gigas effect observed in some cell types such as stomata or pollen grains (Abdoli et al., 2013; Zhang et al., 2016; Salma et al., 2017; Williams and Oliveira, 2020). It happens once chromosomal duplication can cause an increase in the size and volume of the cell, as well as of some reproductive or vegetative organs, such as flowers, anthers, leaves, and seeds (Abdoli *et al*., 2013; Salma *et al*., 2017; Williams and Oliveira 2020). Since pollen size is a potential indirect predictor of ploidy level (Salma *et al*., 2017), we hypothesized that the polar and equatorial axis measurements in *H. lahue* cytotypes would increase with ploidy level. Effectively, our results showed that for *H. lahue* pollen grains, diploids differ significantly from polyploids considering polar and equatorial axis measurements, and polyploids have larger pollen grains compared to diploid samples, as observed in *S. micranthum* (Tacuatiá et al., 2012) and *Sisyrinchium sellowianum* Klatt (Fachinetto et al., 2017). Otherwise, axis measurements are not helpful to determine indirectly the ploidy level in *H. lahue* polyploids, as there are no significant differences in the size of pollen grains between hexaploids and octoploids. 

Our study also detected a significant difference in the amount of pollen per anther between diploids and polyploids, but not between hexaploids and octoploids. Our morphometric data displayed tiny anthers in both polyploids, and this seems related to the reduced amount of the large pollen grains of hexaploids and octoploids of *H. lahue*. Polyploids may have a reduced pollen viability (Tulay and Unal, 2010; Zhang et al., 2016), but the present study found appreciable viability in *H. lahue* pollen samples across all three studied ploidy levels, as observed in other *Herbertia* species, such as the diploid *H. darwinii*, and in the tetraploids *H. pulchella* and *H. quareimana* (Moraes et al., 2015; Stiehl-Alves et al., 2017). Noticeable viability of pollen grains was also detected in other Iridaceae (Tacuatiá et al., 2012; Moraes *et al*., 2015; Fachinetto et al., 2017; Alencar et al., 2018; Burchardt et al., 2018). 

The high pollen viability found for hexaploid and octoploid cytotypes in our study suggests that both have regular meiotic behavior. Although the assessment of pollen viability using colorimetric methods may not have the same efficiency as pollen germination tests, the Alexander staining has allowed indirectly evaluate the meiotic regularity with good safety in several species of Iridaceae. Moraes et al. (2015) analyzed meiotic regularity, meiotic index and pollen viability in 11 species from six genera of Tigridieae. Concerning the polyploid species *H. lahue* and *H. pulchella*, they observed a pollen stainability greater than 98%. In the case of the second species, the meiotic regularity was 99.3% and the meiotic index was 100%, confirming the high rate of pollen viability. According to the authors, although no meiotic analysis was performed for *H. lahue*, its high pollen stainability indicates regular meiosis.

Reproductive data reinforces the regularity in the meiotic behavior of polyploids of *H. lahue*. Stiehl-Alves et al. (2016) studied the breeding system of both polyploid cytotypes of *H. lahue* through hand pollination experiments. They observed in hexaploids a pollination success of 97% and 100%, for self-pollination and cross-pollination, respectively, while octoploids presented 100% of pollination success in both pollination tests. Martins et al. (2021) evaluated seeds traits and germination requirements of three Iridaceae species, including *H. lahue*. Such study showed high seed viability for all ploidy levels. Interestingly, the polyploids *H. lahue* present heavier seeds and the better germination performances than diploids. The whole genome duplications in new polyploids generally result in irregular meiosis and low fertility. Our data and those obtained by Stiehl-Alves *et al*. (2016) and Martins *et al*. (2021) indicate that *H. lahue* polyploids have meiotic stability and high fertility. Taking into account these data, we can suggest that such polyploids go through a period of time sufficient to result in meiotic stability with fertility reestablished and equivalent to the putative diploid parental.

### The effect of polyploidy on morphometric data of *Herbertia lahue*

Our morphometric data grouped *H. lahue* into three clusters corresponding to the cytotypes, with some overlap in polyploid samples. These results agreed with a previous morphometric study on *H. lahue* polyploids (Stiehl-Alves et al., 2016) and highlighted noteworthy differences in androecium and gynoecium characters between cytotypes. Such phenotypic differences between cytotypes, reinforcing the importance of polyploidy as an evolutionary force are remarkable in many plant groups including Iridaceae (Vichiato et al., 2014; Fachinetto et al., 2017; Zenil-Ferguson et al., 2017; Rezende et al., 2020). 

It is recognized that shifts in floral morphology can cause cytotypes to develop distinct life-history traits, since some floral attributes, such as flower size or herkogamy can favor changes in breeding systems (Opedal, 2018; Rezende et al., 2020). Two floral traits analyzed (anther length and style arms free portion length) are particularly relevant for the lack of herkogamy in *H. lahue* polyploids, that are autogamous and capable of self-pollination without pollinators (Stiehl-Alves et al., 2016). Distinct from autogamous *H. lahue* polyploids, herkogamy is discernible in flowers from diploid samples, as evidenced by the morphometric data. 

As in other studies (Vichiato et al., 2014; Zenil-Ferguson et al., 2017; Fachinetto et al., 2017), the gigas effect, was observed in morphometric data of ovaries and underground bulbs from polyploids. A previous study about *H. lahue* analyzed the effect of underground bulb size on flowering characteristics and natural multiplicative capacity and found that plants with larger bulbs produce more flowers with better quality (Morales et al., 2009). The same study observed that *H. lahue* has a low natural multiplicative capacity compared with other commercial geophyte species, but this trait was not associated with the size of the underground bulbs.

Considering phenetic criteria of species boundaries (De Queiroz, 2007), our morphometric analysis partially supports the recent taxonomic classification proposed by Deble (2021), where *H. lahue* was segregated into three species, namely *H. lahue, H. amoena* Grisebach, and *H. caerulea* (Herbert) Herbert. The multivariate analysis identified a group containing 100% of correctly assigned diploid samples, which morphologically correspond to *H. caerulea sensu* Deble. However, hexaploids (morphologically corresponding to *H. amoena sensu* Deble) and octoploids (related to *H. lahue sensu* Deble) were only partially separated by multivariate analysis and thus cannot be considered as distinct species based on phenetic species criteria. 

## Concluding Remarks

This study examines the phenotipic variation in three cytotypes of *H. lahue*. Cytogenetic analysis revealed distinct karyotypic characteristics for each cytotype, corresponding to specific morphotype. Differences were observed between diploids and polyploids regarding morphometric traits, as well as DNA content and the size and quantity of pollen grains, while hexaploids and octoploids revealed fewer distinctions. This is the first time that cytotypes have been compared in a multidisciplinary context, and the results allowed some inferences regarding specific boundaries in *H. lahue,* considering phenetic criteria. This issue can be better understood by testing reproductive isolation and niche modeling, which are potential for delimiting species by responding to biological and ecological criteria. We are currently conducting further research under a multidisciplinary frame, a strategy that is being useful in improving understanding of the evolution of the *H. lahue* complex.

## Supplementary material

The following online material is available for this article:

Table S1 *-*
Geographic detailing and analyses performed in *Herbertia lahue*.

Table S2 *-*
Descriptive statistics of morphological characters examined in *Herbertia lahue*.

Table S3 *-*
Description of results from pollen grain analysis performed in *Herbertia lahue*.

Figure S1 *-*
Inner and outer tepals of *Herbertia lahue* cytotypes.
