# Supplementary material for: Can ploidy levels explain the variation of Herbertia lahue (Iridaceae)?
Source: Genet Mol Biol. 2024 Aug 23;46(3 Suppl 1):e20230137. doi: 10.1590/1678-4685-GMB-2023-0137 (PMC11390242; doi:10.1590/1678-4685-GMB-2023-0137)
Supplement: Table S1 - [file 1415-4757-GMB-46-03-s1-e20230137-s1.pdf]

Genetics and Molecular Biology
https://doi.org/10.1590/1678-4685-GMB-2023-0137

Supplementary Material to "Can ploidy levels explain the variation of *Herbertia lahue* (Iridaceae)?"

Table S1 – Geographic detailing and analyses performed in 38 populations of *Herbertia lahue* sampled in Rio Grande do Sul, Brazil.

Collector (voucher)	Collection	Ploidy level	Locality	Latitude	Longitude	Performed analyzes
Cristante A, 1 (C01)	Living plant	Diploid	Porto Alegre	-30.0676	-51.1198	Pollen analysis
Cristante A, 2 (C02)	Living plant	Diploid	Porto Alegre	-30.0674	-51.1194	Pollen analysis
Cristante A et al., 1 (CMGAA01)	Living plant	Diploid	Guaiba	-30.1801	-51.3926	Pollen analysis
Cristante A et al., 1 (CBS01)	Living plant	Diploid	Porto Alegre	-30.1260	-51.2293	Pollen analysis
Kaltchuk-Santos E et al., 5 (KSAV5)	ICN 202811	Diploid	São Jerônimo do Sul	-30.7430	-51.5120	Cytogenetic
Kaltchuk-Santos E et al., 8 (KSAV8)	ICN 202814	Diploid	São Jerônimo do Sul	-30.7430	-51.5120	Cytogenetic – genome size
Kaltchuk-Santos E et al., 17 (KSAV17)	ICN 202822	Diploid	São Jerônimo do Sul	-30.1048	-51.2333	Cytogenetic – genome size
Stiehl-Alves EM et al., 137 (SATVCH9)	ICN 202838	Diploid	Canguçu	-31.1706	-53.2600	Cytogenetic – morphometry
Stiehl-Alves EM et al., 142 (SATVCH14)	Living plant	Diploid	Canguçu	-31.1649	-52.4204	Morphometry
Stiehl-Alves EM et al., 145 (SATVCH17)	ICN 202842	Diploid	Canguçu	-30.4137	-53.2335	Cytogenetic – morphometry
Stiehl-Alves EM et al., 149 (SATVCH21)	ICN 202844	Diploid	Caçapava do Sul	-30.4959	-53.3014	Morphometry
Stiehl-Alves EM et al., 162 (SATVCH34)	ICN 202846	Diploid	Santana da Boa Vista	-30.4520	-53.9152	Cytogenetic – genome size – morphometry
Stiehl-Alves EM et al., 171 (SATVCH43)	ICN 202847	Diploid	Lavras do Sul	-30.4661	-53.5527	Cytogenetic – morphometry
Cristante A, Marchioretto, RM, 1 (CM01)	Living plant	Hexaploid	Porto Alegre	-30.0713	-51.1192	Pollen analysis
Cristante A, Marchioretto, RM, 2 (CM02)	Living plant	Hexaploid	Porto Alegre	-30.0722	-51.1184	Pollen analysis
Cristante A, 3 (C03)	Living plant	Hexaploid	Porto Alegre	-30.0309	-51.1703	Pollen analysis
Kaltchuk-Santos E et al., 6 (KSAV6)	ICN 202812	Hexaploid	São Jerônimo do Sul	-30.7430	-51.5120	Cytogenetic – genome size
Stiehl-Alves EM et al., 103 (SATVC1)	ICN 202823	Hexaploid	Ipê	-29.1891	-52.2280	Genome size – morphometry
Stiehl-Alves EM et al., 105 (SATVCM2)	ICN 202825	Hexaploid	Santo Antônio das Missões	-28.3242	-54.3319	Cytogenetic – morphometry
Stiehl-Alves EM et al., 108 (SATVCM5)	Living plant	Hexaploid	Santo Antônio das Missões	-28.3022	-55.1347	Morphometry
Stiehl-Alves EM et al., 110 (SATVCM7)	ICN 202827	Hexaploid	Santo Antônio das Missões	-28.3344	-55.9373	Cytogenetic – genome size – morphometry
Stiehl-Alves EM et al., 117 (SATVCM14)	ICN 202830	Hexaploid	Itaqui	-29.0227	-56.3387	Cytogenetic – genome size – morphometry
Stiehl-Alves EM et al., 124 (SATVCM21)	ICN 202833	Hexaploid	Alegrete	-29.5051	-55.5212	Morphometry
Stiehl-Alves EM et al., 172 (SATVCH44)	ICN 202848	Hexaploid	Lavras do Sul	-30.4661	-53.5527	Cytogenetic – morphometry

Collector (voucher)	Collection	Ploidy level	Locality	Latitude	Longitude	Performed analyzes
Cristante, A et al., 07 (CSAM07)	ICN 200589	Octoploid	Caçapava do Sul	-30.8953	-53.4303	Pollen analysis
Cristante, A et al., 09 (CSAM09)	ICN 200587	Octoploid	Caçapava do Sul	-30.8936	-53.4292	Pollen analysis
Kaltchuk-Santos E et al., 3 (KSAV3)	ICN 202810	Octoploid	São Jerônimo do Sul	-30.8553	-51.5420	Cytogenetic
Stiehl-Alves EM et al., 104 (SATVCM1)	ICN 202824	Octoploid	São Miguel das Missões	-28.3242	-54.3319	Cytogenetic – morphometry
Stiehl-Alves EM et al., 111 (SATVCM8)	ICN 202828	Octoploid	Santo Antônio das Missões	-28.3344	-55.9373	Cytogenetic – morphometry
Stiehl-Alves EM et al., 115 (SATVCM12)	ICN 202829	Octoploid	Itaqui	-28.5951	-56.0550	Cytogenetic – morphometry
Stiehl-Alves EM et al., 118 (SATVCM15)	ICN 202831	Octoploid	Itaqui	-29.0227	-56.3387	Morphometry
Stiehl-Alves EM et al., 123 (SATVCM20)	ICN 202832	Octoploid	Alegrete	-29.5051	-55.5212	Cytogenetic – genome size – morphometry
Stiehl-Alves EM et al., 129 (SATVCH1)	ICN 202835	Octoploid	Cristal	-30.5435	-52.4224	Cytogenetic – morphometry
Stiehl-Alves EM et al., 132 (SATVCH4)	ICN 202836	Octoploid	Canguçu	-31.2148	-52.4448	Genome size – morphometry
Stiehl-Alves EM et al., 134 (SATVCH6)	ICN 202837	Octoploid	Canguçu	-31.2144	-52.4826	Cytogenetic
Stiehl-Alves EM et al., 138 (SATVCH10)	ICN 202839	Octoploid	Canguçu	-31.1706	-53.2600	Morphometry
Stiehl-Alves EM et al., 148 (SATVCH20)	ICN 202843	Octoploid	Caçapava do Sul	-30.4959	-53.3014	Cytogenetic – genome size – morphometry
Stiehl-Alves EM et al., 161 (SATVCH33)	ICN 202845	Octoploid	Santana da Boa Vista	-30.4520	-53.9152	Cytogenetic – morphometry
