# Supplementary material for: Can ploidy levels explain the variation of Herbertia lahue (Iridaceae)?
Source: Genet Mol Biol. 2024 Aug 23;46(3 Suppl 1):e20230137. doi: 10.1590/1678-4685-GMB-2023-0137 (PMC11390242; doi:10.1590/1678-4685-GMB-2023-0137)
Supplement: Table S2 - [file 1415-4757-GMB-46-03-s1-e20230137-s2.pdf]

Genetics and Molecular Biology
https://doi.org/10.1590/1678-4685-GMB-2023-0137

# Supplementary Material to "Can ploidy levels explain the variation of *Herbertia lahue* (Iridaceae)?"

**Table S2 –** Descriptive statistics of 20 characters examined in diploid, hexaploid and octoploid *Herbertia lahue* cytotypes.

| **Parameters**[1] | **Diploid** | | | | **Hexaploid** | | | | **Octoploid** | | | | **Statistics** | | |
|---|---|---|---|---|---|---|---|---|---|---|---|---|---|---|---|
| | ***Mean (± SD)** | **Min** | **Max** | **N** | **Mean (± SD)** | **Min** | **Max** | **N** | **Mean (± SD)** | **Min** | **Max** | **N** | ***F*** | **DF** | ***P*-value** |
| Bulb length | 22.1 ± 4.0 b | 12.0 | 31.0 | 43 | 22.5 ± 5.6 b | 12.0 | 34.0 | 79 | 25.9 ± 4.9 a | 13.0 | 40.0 | 79 | 11.1 | 150 | 3.1 × 10-5 *** |
| Bulb width in minor axis | 10.6 ± 2.1 b | 7.0 | 15.0 | 42 | 11.7 ± 2.6 b | 6.0 | 16.0 | 78 | 14.6 ± 3.2 a | 8.0 | 23.0 | 78 | 30.7 | 148 | $6.8 \times 10^{-12}$ *** |
| Bulb width in major axis | 11.4 ± 2.3 c | 7.0 | 15.0 | 42 | 13.1 ± 2.4 b | 8.0 | 1.7 | 78 | 15.5 ± 3.3 a | 9.0 | 23.0 | 78 | 29.7 | 148 | $1.4 \times 10^{-11}$*** |
| Ovary length | 6.6 ± 0.5 b | 5.5 | 7.6 | 74 | 7.5 ± 1.0 a | 5.3 | 9.0 | 164 | 7.9 ± 0.9 a | 6.0 | 10.9 | 64 | 51.9 | 325 | $< 2.2 \times 10^{-16}$ *** |
| Ovary width | 2.5 ± 0.3 b | 1.7 | 3.0 | 75 | 2.6 ± 0.2 ab | 2.0 | 3.0 | 165 | 2.6 ± 0.4 a | 2.0 | 3.9 | 165 | 6.9 | 324 | 0.0 ** |
| Outer tepal length | 26.4 ± 2.7 a | 21.0 | 32.0 | 75 | 21.3 ± 1.9 b | 17.0 | 24.5 | 165 | 20.8 ± 1.8 b | 17.1 | 24.9 | 165 | 196.2 | 327 | $< 2.2 \times 10^{-16}$ *** |
| Outer tepal width 1 | 2.6 ± 0.6 | 1.7 | 3.9 | 75 | 2.6 ± 0.5 | 1.1 | 3.9 | 165 | 2.6 ± 0.9 | 1.1 | 4.8 | 165 | 0.3 | 327 | 0.8 ns |
| Outer tepal width 2 | 5.2 ± 0.3 a | 4.1 | 5.6 | 75 | 4.6 ± 0.4 b | 3.8 | 5.9 | 154 | 5.2 ± 1.1 a | 2.7 | 7.6 | 154 | 13.8 | 311 | $1.9 \times 10^{-6}$ *** |
| Outer tepal width 3 | 20.1 ± 1.2 a | 19.1 | 25.0 | 60 | 6.1 ± 0.8 c | 4.3 | 7.8 | 165 | 10.0 ± 2.4 b | 4.9 | 14.8 | 165 | 1068.0 | 312 | $< 2.2 \times 10^{-16}$ *** |
| Outer tepal width 4 | 20.1 ± 0.5 a | 19.2 | 20.9 | 60 | 9.4 ± 1.8 c | 6.1 | 12.4 | 165 | 11.6 ± 3.1 b | 7.1 | 22.0 | 165 | 361.2 | 312 | $< 2.2 \times 10^{-16}$ *** |
| Inner tepal length | 7.2 ± 0.6 b | 6.0 | 9.0 | 74 | 7.9 ± 0.6 a | 7.2 | 9.1 | 165 | 6.8 ± 0.7 c | 5.1 | 8.8 | 165 | 78.4 | 326 | $< 2.2 \times 10^{-16}$ *** |
| Inner tepal width | 2.5 ± 0.3 b | 2.2 | 2.9 | 75 | 3.0 ± 0.4 a | 2.4 | 3.9 | 159 | 2.4 ± 0.5 bc | 1.7 | 3.9 | 159 | 53.5 | 321 | $< 2.2 \times 10^{-16}$ *** |
| Stamens anther length | 7.5 ± 0.6 a | 6.0 | 8.9 | 72 | 4.6 ± 0.4 c | 4.0 | 5.6 | 165 | 5.3 ± 0.8 b | 4.1 | 7.5 | 165 | 24.1 | 306 | $1.9 \times 10^{-10}$ *** |
| Stamens anther width | 1.1 ± 0.3 a | 0.6 | 1.8 | 75 | 0.8 ± 0.1 c | 0.6 | 1.0 | 150 | 0.9 ± 0.3 b | 0.4 | 2.0 | 150 | 56.1 | 327 | $< 2.2 \times 10^{-16}$ *** |
| Stamens connate portion | 5.0 ± 0.5 a | 4.0 | 6.0 | 75 | 4.2 ± 0.4 c | 3.1 | 4.9 | 165 | 4.6 ± 0.6 b | 3.0 | 5.8 | 165 | 408.7 | 327 | $< 2.2 \times 10^{-16}$ *** |
| Stamens adnate portion | 3.6 ± 0.4 a | 3.0 | 4.7 | 66 | 2.8 ± 0.4 b | 2.0 | 3.6 | 148 | 2.6 ± 0.5 c | 1.1 | 4.0 | 148 | 91.7 | 301 | $< 2.2 \times 10^{-16}$ *** |
| Style total lenght | 4.2 ± 0.5 a | 3.0 | 5.1 | 75 | 3.2 ± 0.5 b | 2.1 | 3.8 | 165 | 2.8 ± 0.4 c | 2.0 | 3.8 | 165 | 253.2 | 327 | $< 2.2 \times 10^{-16}$ *** |
| Style arms total length | 1.8 ± 0.3 a | 1.1 | 2.3 | 74 | 0.7 ± 0.2 b | 0.6 | 1.4 | 164 | 0.7 ± 0.2 b | 0.4 | 1.5 | 164 | 635.9 | 318 | $< 2.2 \times 10^{-16}$ *** |
| Style arms concrescent portion | 1.9 ± 0.6 | 1.1 | 2.9 | 75 | 2.0 ± 0.5 | 1.0 | 3.3 | 165 | 2.0 ± 0.5 | 1.0 | 3.3 | 165 | 1.9 | 320 | 0.1 ns |
| Style arms free portion | 2.3 ± 0.4 a | 1.5 | 2.9 | 73 | 1.4 ± 0.7 b | 0.2 | 2.8 | 156 | 0.7 ± 0.3 c | 0.2 | 1.3 | 156 | 326.6 | 311 | $< 2.2 \times 10^{-16}$ *** |

*Notes*: [1] The measurements are described in millimeters. N indicates sample number, Min indicates minimum values observed, Max indicates maximum values observed, DF indicates degrees of freedom. *Mean values ± SD (standard deviation) in lines followed by a different letter(s) are significantly different at $p \leq 0.05$ according to Tukey or Duncan's test. Levels of significance of *F*-statistics were $P > 0.05$: not significant (ns); $P \leq 0.01$: very significant (**); $P \leq 0.001$: highly significant (***).
