# Supplementary material for: Can ploidy levels explain the variation of Herbertia lahue (Iridaceae)?
Source: Genet Mol Biol. 2024 Aug 23;46(3 Suppl 1):e20230137. doi: 10.1590/1678-4685-GMB-2023-0137 (PMC11390242; doi:10.1590/1678-4685-GMB-2023-0137)
Supplement: Table S3 - [file 1415-4757-GMB-46-03-s1-e20230137-s3.pdf]

Genetics and Molecular Biology
https://doi.org/10.1590/1678-4685-GMB-2023-0137

Supplementary Material to "Can ploidy levels explain the variation of *Herbertia lahue* (Iridaceae)?"

Table S3 – Description of results for pollen grain analysis performed in diploid, hexaploid and octoploid *Herbertia lahue* cytotypes.

**Parameters**	**Diploid**	**Hexaploid**	**Octoploid**
Equatorial axis (µm)			
Number of pollen grains measured	360	320	180
Mean	36.6 [b]	46.5 [a]	46.8 [a]
Standard deviation	± 4.0	± 6.4	± 8.2
Minimum values	26.7	30.4	30.9
Maximum values	53.5	58.6	64.9
Polar axis (µm)			
Number of pollen grains measured	360	320	180
Mean	34.2 [b]	40.4 [a]	39.8 [a]
Standard deviation	± 3.2	± 4.8	± 5.6
Minimum values	24.5	28	30.4
Maximum values	47.4	56.6	56.2
Pollen grain quantification			
Number of plants sampled	18	16	10
Mean of pollen grains per flower	58694.2 [a]	23656.2 [b]	26791.7 [b]
Standard deviation	± 9748.8	± 20676.1	± 9766.3
Minimum values	44166	11750	18375
Maximum values	70555	54625	37500
Mean of pollen grains per anther	19564.4 [a]	7885.0 [b]	8930.3 [b]
Standard deviation	± 3249.6	± 6892.1	± 3255.5
Minimum values	44166	11750	18375
Maximum values	70555	54625	37500
Pollen viability			
Percentage of viable pollen grains	94%	94%	93%
Number of viable pollen grains	8465	7485	4186
Mean of viable pollen grains	1693.0	1871.3	1395.3
Standard deviation	274.0	85.0	510.0
Minimum values	1356	1805	973
Maximum values	1930	1989	1962
Number of non-viable pollen grains	535	515	314

[a, b] Letters indicate differences and mean values marked with the same letter are not significantly different at $P < 0.05$, by Tukey's test.
