# Supplementary material for: Can ploidy levels explain the variation of Herbertia lahue (Iridaceae)?
Source: Genet Mol Biol. 2024 Aug 23;46(3 Suppl 1):e20230137. doi: 10.1590/1678-4685-GMB-2023-0137 (PMC11390242; doi:10.1590/1678-4685-GMB-2023-0137)
Supplement: Figure S1 - [file 1415-4757-GMB-46-03-s1-e20230137-s4.pdf]

Genetics and Molecular Biology
https://doi.org/10.1590/1678-4685-GMB-2023-0137

# Supplementary Material to "Can ploidy levels explain the variation of *Herbertia lahue* (Iridaceae)?"

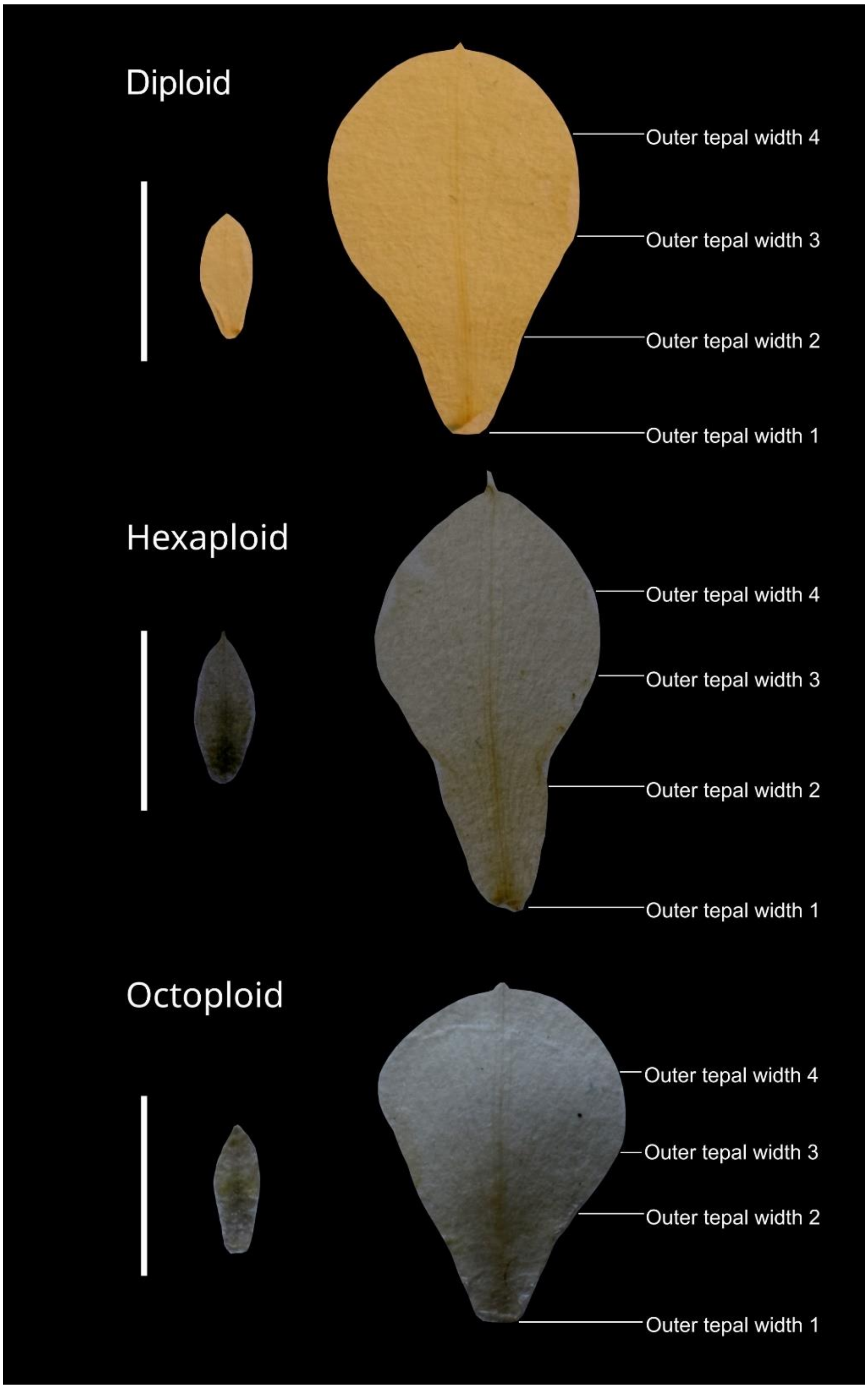


**Figure S1** – Inner and outer tepals of *Herbertia lahue* cytotypes. Bars: 1 cm.
